# Integrated complex care coordination for children with medical complexity: A mixed-methods evaluation of tertiary care-community collaboration

**DOI:** 10.1186/1472-6963-12-366

**Published:** 2012-10-23

**Authors:** Eyal Cohen, Ashley Lacombe-Duncan, Karen Spalding, Jennifer MacInnis, David Nicholas, Unni G Narayanan, Michelle Gordon, Ivor Margolis, Jeremy N Friedman

**Affiliations:** 1 Department of Paediatrics, The Hospital for Sick Children, University of Toronto, 555 University Avenue, Toronto M5G 1X8, ON, Canada; 2 Institute of Health Policy, Management & Evaluation, University of Toronto, 155 College Street, Suite 425, Toronto M5T 3M6, ON, Canada; 3 CanChild Centre for Childhood Disability Research, McMaster University, 1400 Main Street West, Room 408, Hamilton, L8S 1C7, ON, Canada; 4 Daphne Cockwell School of Nursing, Ryerson University, 350 Victoria Street, Toronto, M5B 2K3, ON, Canada; 5 Faculty of Social Work, University of Calgary, 2500 University Drive NW, Calgary, T2N 1N4, AB, Canada; 6 Department of Orthopedic Surgery, The Hospital for Sick Children, University of Toronto, 555 University Avenue, Toronto, M5G 1X8, ON, Canada; 7 Soldiers Memorial Hospital, 170 Colborne Street West, Orillia, L3V 2Z3, ON, Canada; 8 Brampton Civic Hospital, 150 Central Park Drive, Brampton, L6T 2T9, ON, Canada; 9 Bloorview Research Institute, Holland Bloorview Kids Rehabilitation Hospital, 150 Kilgour Road, Toronto, M4G 1R8, ON, Canada

**Keywords:** Complex care, Care coordination, Special needs, Integration

## Abstract

**Background:**

Primary care medical homes may improve health outcomes for children with special healthcare needs (CSHCN), by improving care coordination. However, community-based primary care practices may be challenged to deliver comprehensive care coordination to complex subsets of CSHCN such as children with medical complexity (CMC). Linking a tertiary care center with the community may achieve cost effective and high quality care for CMC. The objective of this study was to evaluate the outcomes of community-based complex care clinics integrated with a tertiary care center.

**Methods:**

A before- and after-intervention study design with mixed (quantitative/qualitative) methods was utilized. Clinics at two community hospitals distant from tertiary care were staffed by local community pediatricians with the tertiary care center nurse practitioner and linked with primary care providers. Eighty-one children with underlying chronic conditions, fragility, requirement for high intensity care and/or technology assistance, and involvement of multiple providers participated. Main outcome measures included health care utilization and expenditures, parent reports of parent- and child-quality of life [QOL (SF-36®, CPCHILD^©^, PedsQL™)], and family-centered care (MPOC-20®). Comparisons were made in equal (up to 1 year) pre- and post-periods supplemented by qualitative perspectives of families and pediatricians.

**Results:**

Total health care system costs decreased from median (IQR) $244 (981) per patient per month (PPPM) pre-enrolment to $131 (355) PPPM post-enrolment (p=.007), driven primarily by fewer inpatient days in the tertiary care center (p=.006). Parents reported decreased out of pocket expenses (p<.0001). Parental QOL did not significantly change over the course of the study. Child QOL improved between baseline and 6 months in two PedsQL™ domains [Social (p=.01); Emotional (p=.003)], and between baseline and 1 year in two CPCHILD^©^ domains [Health Standardization Section (p=.04); Comfort and Emotions (p=.03)], while total CPCHILD^©^ score decreased between baseline and 1 year (p=.003). Parents and providers reported the ability to receive care close to home as a key benefit.

**Conclusions:**

Complex care can be provided in community-based settings with less direct tertiary care involvement through an integrated clinic. Improvements in health care utilization and family-centeredness of care can be achieved despite minimal changes in parental perceptions of child health.

## Background

By improving care coordination, primary care medical homes may improve health outcomes for children with special healthcare needs (CSHCN). These are children who have a chronic physical, developmental, behavioral or emotional condition and who also require health and related services of a type or amount beyond that required by children generally [1,2]. However, primary care practices based in the community may be challenged to deliver comprehensive care coordination to complex subsets of CSHCN such as children with medical complexity (CMC), defined as those children with substantial family-identified needs, characteristic complex and/or chronic conditions, functional limitations, and high health care use [3]. In recent years, children’s hospitals have experienced a documented growth in the relative numbers and costs related to CMC who receive care [4,5]. While CMC represent a small proportion of all pediatric health care consumers, they have disproportionately high acute care utilization [4,6]. Consequently, some children’s hospitals have developed structured complex care programs that provide comprehensive care delivery (either as primary care providers (PCPs) or as a co-management model with community-based PCPs) for CMC [7-11].

Complex care programs structurally located in tertiary care centers offer important benefits, including access to various specialists and services, and the ability to provide ‘one-stop’ care for CMC. However, for families living far from such hospitals, traveling repeatedly for care can be disruptive. Further, providers in tertiary care hospitals may lack knowledge of community-based services, limiting their ability to provide comprehensive care coordination. Population-specific, community-oriented primary care integrated with flexible hospital services, is expected to contribute to accessible, comprehensive, continuous and patient-centred health care [12]. Linking a tertiary care center to ‘community based’ practices through an integrated practice model may play an important role in providing cost effective and high quality care for CMC. The objective of this study was to evaluate the effectiveness of a novel community–based complex care clinic integrated with a tertiary care facility.

## Methods

### Study design

The study was designed as a before- and after- intervention study design using mixed (quantitative/qualitative) methods.

### Study setting

The study was conducted from December, 2008 to December, 2010 in two community general hospitals within the catchment area of a large tertiary care children’s hospital, The Hospital for Sick Children (SickKids) in Toronto, Ontario, Canada. Brampton Civic Hospital (608 beds) is located in Brampton, Ontario, with a population of approximately half a million, located approximately 40 kilometers from SickKids. Orillia Soldier’s Memorial Hospital (230 beds) is located in Orillia, Ontario, with a population of fifty thousand, located approximately 130 kilometers from SickKids. In common, both sites are busy community hospitals staffed primarily by community-based consultant pediatricians, and utilize SickKids for much of their subspecialty care. Brampton serves a suburban catchment population of whom 60% are visible minorities (most commonly of South Asian or Afro-Caribbean descent), while Orillia serves a large rural catchment of mainly Caucasians extending into areas of Northern Ontario, which are many hours away by car from SickKids.

### Participants

Referrals for the study were made by local primary care physicians based on criteria that are common in structured complex care programs [7] and reflected a recently-developed conceptual definition of medical complexity [3]. Specific inclusion criteria were: (A) children (<16 years) with a known and/or suspected diagnosis of a complex chronic condition that is associated with medical fragility; (B) technology assistance (e.g. gastrostomy tube, tracheostomy tube) *or* the need for as high an intensity of care as a technology assisted child; and, (C) involvement of multiple specialists, such as gastroenterologists, neurologists, etc., including at least one at SickKids. Families referred were also, in the opinion of the referring physician, thought to have unmet needs. We only included children of families who were able to communicate in English or Punjabi, the most common non-English language spoken in Brampton. Excluded were children enrolled in a disease-specific care program (e.g. cystic fibrosis) or those already enrolled in a previously described complex care program situated at SickKids [13]. Patients referred to the complex care program at SickKids who lived within the catchment of the two participating community hospitals were invited to participate in the study in lieu of care through the SickKids complex care clinic. Informed consent and, where applicable, child assent was collected for all participants. The study protocol was approved by the Hospital for Sick Children Research Ethics Board (REB# 1000013191), Orillia Soldiers’ Memorial Hospital Institutional Review Board and the William Osler Health Centre Research Ethics Board.

### Intervention

The clinic was run as a co-management model with existing primary care providers (PCPs). Clinics were staffed by local community pediatricians (4 per site), together with a tertiary care affiliated nurse practitioner (NP) (0.5 full time equivalent per site), recruited specifically for this project. NPs were pediatric nurse practitioners with advanced skills and education (Master’s degree) in the provision of care to CMC, direct previous clinical experience working with CMC, and comprehensive knowledge and understanding of various disease processes and effects on children and families. Patients were referred from their existing local PCP (family doctor or primary-care pediatrician; in Canada, primary pediatric care is provided by either type of physician) who remained involved in important aspects of primary care such as immunizations and visits for inter-current illnesses. Clinics were conducted weekly at each site and the NP participated via telemedicine during anticipated periods of inclement weather (winter). The focus of visits was on care coordination (e.g. coordinating multiple subspecialty consultations or ensuring availability of equipment from home health care agencies), complex symptom management (e.g. complex feeding problems and/or respiratory issues), and goal setting (e.g. advanced directives). A care plan was developed by the NP in partnership with the family for all patients, using an electronic template created specifically for this project with information such as goals of care, patient-specific emergency management guidelines, updated medication lists, itemized medical issues, and the names and contact details of the health care team. This care plan was given to the families and also uploaded to the province-wide e-portal [the electronic Child Health Network (eCHN)] so that summative information would be available to providers across the continuum. Allied health support from a social worker and dietician was available to the clinic when necessary, and other community-based providers (e.g. home care nurses and/or case managers and teachers) were encouraged to attend as well. Community-based therapists and professional support services contributing to a holistic circle of care for CMC that may have been accessed by NPs (dependent on care needs of patients and resources available in the community) have been described in a previous publication [14]. The NP also had access to hospital-based pediatricians (pediatric hospitalists) from the complex care program at SickKids (n=8) for further consultative support, although they did not play a direct role in the patients’ care. Communication by the family through email and telephone with the NP was encouraged.

### Outcomes

#### Evaluative framework

Evaluation was structured around the Institute for Healthcare Improvement (IHI) Triple Aim including measures that evaluate improvements in the health of a population, individual experiences of care, and per capita cost (Table 1) [15].

**Table 1:** **Measures of Health, Experience and Cost Utilizing the Institute for Healthcare Improvement (IHI) Triple Aim**[15]

**Element of the IHI Triple Aim**[15]	**Measures**
**Health of a Population**	Parental reports of health-related quality of life (SF-36®)
	Child Health-Related Quality of Life (PedsQL™)
	Caregiver Priorities and Child Health Index of Life with Disabilities (CPCHILD®)
	*Qualitative Interviews*
**Individual Experience of Care**	Parental Perceptions of Family-Centreedness of Care (MPOC^©^)
	*Qualitative Interviews*
**Per Capita Cost**	Health and Social Service Utilization Questionnaire
	Inpatient days,
	Hospital-based costs (ER, inpatient, outpatient)
	[total cost per patient per month (PPPM)]

ER = emergency room.

### Data collection

The child’s primary caregiver, defined as the single person most responsible for the day-to-day care and decision-making for the child, participated in semi-structured interviews at recruitment, 6 months, and 1 year of follow-up with a research coordinator who was not involved in direct patient care. In this study, the caregivers were either natural parents of the children (n=68), or classified as foster, adoptive parents, or grandparents (n=13).

### Measures

Family income, occupation, and education level were collected using standardized questions [16]. Primary caregivers also completed a structured questionnaire about their children’s medical problems, medications, technology assistance, hospitalizations, emergency department (ED) visits as well as visits to hospital- and community-based practitioners. Follow-up questionnaires on health resource utilization were administered to parents at 6 months and 12 months. Patient charts and hospital databases were abstracted to collect supplementary clinical information such as specific diagnoses and to fill in any gaps in parental recall. Diagnoses were categorized according to complex chronic conditions (CCCs) and neurologic impairment (NI), utilizing International Classification of Diseases, 9^th^ and 10^th^ revision codes. CCCs were defined based on Feudtner’s list that incorporates any medical condition that can be reasonably expected to last at least 12 months (unless death intervenes) and to involve either several different organ systems or 1 organ system severely enough to require specialty pediatric care and probably some period of hospitalization in a tertiary care center [17]. NI was based on Srivastava’s definition of diagnoses consistent with static or progressive neurological, genetic or other disease that typically results in either functional and/or intellectual impairment [18]. Technology assistance was defined as requiring assistance from technology, such as a tracheostomy tube, feeding tube, or a wheelchair, for activities of daily living.

### Costing

Costing estimates incorporated both third-party payer (Ontario’s Ministry of Health and Long-Term Care) and parental perspectives (out-of-pocket costs). In Ontario, all hospital care and physician visits are covered through provincial health insurance. Medication, home care, and other types of services (e.g. rehabilitation, school-based care, and/or devices) are paid for by one or more of government, private insurers and/or out-of-pocket payment [19]. Hospital- and community-based ambulatory clinic visits days, emergency room visits, day surgery visits and total health-related expenditures (in Canadian dollars) were collected for all patients enrolled in the study for up to one year pre- and one year post-enrollment utilizing standard methodology from the Ontario Case Costing Initiative (OCCI) [20], commonly used in Canadian health services research [21]. Hospitals participating in the OCCI have implemented standardized case costing methodology developed by the OCCI and have participated in activities to ensure data quality. Currently, the OCCI collects case cost data for acute inpatient, day surgery, ambulatory care, complex continuing care, and rehabilitation.

Out-of-pocket costing for health and social services was captured using the Expenditures for Health and Social Service Utilization Questionnaire [22,23], which consists of questions about the respondent’s use of eight categories of direct health services. To calculate annual utilization measures, the various spans of time were adjusted to yield an annual rate of utilization per category of health service [24]. The annual rate per category of service was multiplied by the 2006 unit cost for that service, and adjusted to 2009 levels (the midpoint of the study) using the medical services component of the consumer price index [25].

### Health-related quality of life (HR-QOL)

Parental HR-QOL was assessed using the Medical Outcomes Study 36-item Short Form (SF-36®) [26], administered at baseline and at 12 months. This instrument measures HR-QOL in eight domains with standardized scores ranging from 0–100. Child HR-QOL was assessed with instruments administered to the primary caregiver at baseline, 6 and 12 months. The PedsQL™, a widely used generic measure of HR-QOL, was used in children ≥ 2 years. The Caregiver Priorities & Child Health Index of Life with Disabilities (CPCHILD®) measures the ease of care, comfort, health and well-being of children with severe disabilities [27], typical of many children enrolled in complex care interventions [7]. The CPCHILD^©^ reports standardized scores from 0 – 100 for each of six domains as well as overall health and QOL and was administered for children ≥1 year. Lastly, parental perceptions of care were evaluated using the Measures of Processes of Care (MPOC^©^) [28,29], that measures caregiver perceptions of the extent of family-centeredness of the care received by children with disabilities in a health care organization. The MPOC^©^ is composed of five subscales with scale scores ranging from 1 to 7; higher scores indicate that parents perceive their needs are being better met.

### Interviews/Focus groups

A sample of parents (n=20; 10 per site) purposefully chosen to ensure maximal variation in severity of child conditions, types of care needs as well as parental gender, socio-economic status, culture and educational level were invited to participate in one-time interviews examining their experiences of receiving care. A sample of Health Care Providers (HCPs) including both community physicians who referred patients to the clinic (n=6) as well as those who attended the clinic (n=6) participated in one of two focus groups (one at each site) to elicit feedback on the effect of the Complex Care Clinic on patient care.

### Data analysis

#### Quantitative data

Baseline demographics of children and families, socio-economic status, caregiver health (SF-36), child health/well-being (CPCHILD^©^copy;, PedsQL™), caregiver satisfaction (MPOC^©^), and health care utilization, were summarized using descriptive statistics. For all analyses, the first visit to the clinic was considered the date of enrollment. Change in summary scores over the period of follow-up of parental health expenditures, caregiver health (SF-36®), child health/well-being (CPCHILD^©^, PedsQL™) and caregiver satisfaction (MPOC^©^) were compared using non-parametric analyses (Friedman test); Sidak adjustments were used to adjust the P value for multiple comparisons. Case costing data (from health administrative databases) were compared between equal (up to one year) pre- and post-enrolment using Wilcoxon signed-rank test. Visit number data (from health administrative databases) were compared between equal (up to one year) pre- and post-enrolment using paired t-tests. Full one year of pre- and post- data was unavailable for n=15 participants [birth less than one year pre-enrolment (n=14) or death less than one year post-enrolment (n=1)]. In order to account for this which led to varying number of participants across months, all cost data were standardized on a per patient per month (PPPM) basis and costs were adjusted for inflation. As well, overall costing trends were analyzed using a repeated measures design with a mixed model analysis, within each site (community and tertiary care) and between sites. Data was log transformed to stabilize the variance and model fit was tested using the Akaike Information Criterion (AIC). Differences were considered significant at p<.05. All statistical analyses were performed using SAS 9.3 (SAS Institute Inc., Cary, North Carolina) and Minitab Statistical Software® (Minitab Inc., Pennsylvania, United States).

#### Qualitative data

Interviews and focus groups were audio-recorded, transcribed verbatim, and de-identified. Content analysis of the data from parent interviews and HCP focus groups was structured around the creation of categories [30], and, informed by the IHI framework, helped to provide a better understanding of the effect of the intervention on patient care. Two team members (EC and JM) coded interviews and focus groups independently until meaningful categories emerged. Disagreements were resolved by consensus or by adjudication from another team member (KS). NVivo 9 (QSR International, Cambridge, MA) was utilized for qualitative data management and analysis.

## Results

### Demographic and clinical characteristics

All families participating in the intervention consented to participate in the evaluation. Eighty-one children (42 in Orillia; 39 in Brampton) and their primary care giver (85% female) were recruited (Table 2). One child died of his underlying condition during less than one month into the study. Neurologic impairment, complex chronic conditions (CCCs), and technology assistance (TA) were common in the cohort. The most common diagnostic category among those children with CCCs was neuromuscular conditions (46/77; 60%), followed by respiratory (30/77; 39%) and cardiovascular (27/77; 35%) and the most common form of TA was a gastrostomy tube (32/46; 70%), followed by a wheelchair (17/46; 37%).

**Table 2:** Participant demographics and clinical characteristics among participants (n=81)

**Variable**	
**Male gender, n (%)**	52 (64%)
**Age in years, mean (SD)**	5.8 (4.7)
**Primary caregiver**	
**Mother, n (%)**	69 (85%)
**Gross Household Income, n (%):**	
**$0-29 999**	19 (31%)
**$30,000 – 59 999**	19 (31%)
**$60,000 – 79 999**	5 (8%)
**$80 000 or more**	18 (30%)
**Chronic diagnoses, mean (SD)**	8 (3)
**Specialists seen in previous 6 months, mean (SD)**	4 (2)
**Prescription meds, mean (SD)**	4 (3)
**Complex chronic conditions**^**a**^	77 (95%)
**Neurological impairment, n (%)**^**b**^	78 (96%)
**Technology assistance**^**c**^	46 (57%)

^a^ Using Feudtner’s definition [17] (utilizing International Classification of Diseases, 9^th^ and 10^th^ revision codes) of: any medical condition that can be reasonably expected to last at least 12 months (unless death intervenes) and to involve either several different organ systems or 1 organ system severely enough to require specialty pediatric care and probably some period of hospitalization in a tertiary care center.

^b^ Neurological impairment was based on Srivastava’s definition [18] of diagnoses consistent with static or progressive neurological, genetic or other disease that typically results in either functional and/or intellectual impairment.

^c^ Technology assistance was defined as requiring assistance from technology, such as a tracheostomy tube, feeding tube, or a wheelchair, for activities of daily living.

### Health resource utilization

#### Administrative database (Case costing)

**Figure 1:**
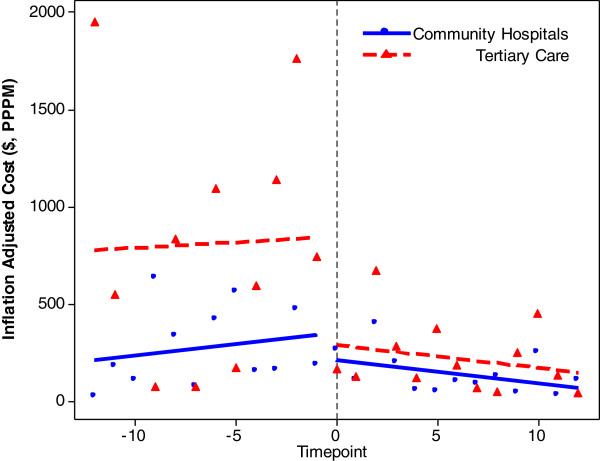
Health-related expenditures per month at tertiary care (SickKids) and community hospitals (Orillia and Brampton).

Overall hospital costs declined over the study period (Figure 1; Table 3). Median (IQR) total costs at the sites combined, decreased from $244 (982) per patient per month (PPPM) pre-enrolment to $131 (355) (p=.007). This occurred despite an increase in overall outpatient costs (p=.0008). The overall decline was driven mainly by a drop in overall inpatient hospital days (p=.0005), particularly at SickKids. Initially most hospitalizations occurred in tertiary care, but by the last six months, the majority of inpatient hospital days were in community hospitals.

**Table 3:** Healthcare utilization for 12 months pre and post-enrolment

	**Cost , $, per patient per month**					**Visits , #, per patient per 12 months**				
	**Pre Mean (SD)**	**Pre Median (IQR)**	**Post Mean (SD)**	**Post Median (IQR)**	*P value	**Pre Mean (SD)**	**Pre Median (IQR)**	**Post Mean (SD)**	**Post Median (IQR)**	**P value
**Total (n=81**^**a**^**)**										
**Overall**	$1439 (3511)	$244 (982)	$369 (708)	$131 (355)	0.007	20 (24)	8 (24)	14 (16)	10 (13)	0.009
**ER**	$23 (35)	$14 (35)	$15 (23)	$0 (19)	0.004	1.35 (1.8)	1 (2)	1 (1.5)	0 (2)	0.05
**Outpatient**^**b**^	$70 (103)	$42 (70)	$82 (64)	$64 (56)	0.0008	6.9 (10.3)	4 (6)	9.0 (6.7)	8 (7)	0.05
**Inpatient**^**c**^	$1343 (3494)	$0 (907)	$273 (677)	$0 (295)	0.0005	11.7 (21.4)	0 (13)	3.7 (11.3)	0 (4)	0.0005
**Tertiary Care**	(SickKids)	(n=81)								
**Overall**	$1145 (3499)	$41 (406)	$230 (629)	$50 (193)	0.07	11 (21)	3 (8)	6 (13)	3 (8)	0.03
**ER**	$11 (27)	$0 (9)	$5 (15)	$0 (0)	0.03	0.5 (1)	0 (1)	0.3 (0.8)	0 (0)	0.08
**Outpatient**	$42 (63)	$20 (59)	$51 (54)	$41 (63)	0.06	2.6 (3.1)	1 (4)	3.8 (3.8)	3 (5)	0.002
**Inpatient**	$1092 (3477)	$0 (311)	$172 (605)	$0 (60)	0.005	8.0 (19.3)	0 (3)	2.3 (10.4)	0 (1)	0.006
**Community Hospitals**	(Orillia and Brampton)	(n=81)								
**Overall**	$293 (659)	$40 (234)	$140 (220)	$39 (183)	0.7	9 (14)	3 (9)	7 (7)	6 (8)	0.24
**ER**	$12 (21)	$0 (14)	$9 (15)	$0 (14)	0.07	0.9 (0.7)	0 (1)	0.7 (1.1)	0 (1)	0.24
**Outpatient**	$28 (64)	$6 (24)	$31 (25)	$23 (28)	<0.0001	4.4 (9.9)	1 (4)	5.3 (4.4)	5 (5)	0.38
**Inpatient**	$252 (653)	$0 (187)	$100 (205)	$0 (125)	0.2	3.8 (9.9)	0 (3)	1.4 (2.8)	0 (2)	0.036

* Statistical analyses for cost using Wilcoxon signed-rank test.

** Statistical analyses for visit number using paired t-test.

ER = emergency room.

^a^ All patients enrolled in the study, cost reflective of all patients, including those who did not utilize services at least once during the study period.

^b^ Outpatient costs and visits are reflected by clinic visits plus day surgery.

^c^ Inpatient visits are reflected by bed days.

#### Parental reports

Overall, parents reported that out-of-pocket expenses declined (p<.0001) (Table 4). In the first six months, expenses increased from a median (IQR) of $813 (2793) per child at baseline to $3111 (4489) (p=.001), but then declined to $538 (2747) per child at 12 months (p=.0001). Over 12 months, medication costs declined (p<.0001), costs associated with work loss due to treatment declined (p=.0006), and costs of diagnostic tests declined (p=.008), while families received more money from government cheques (p<.0001).

**Table 4:** Caregiver-reported Health and Social Service Utilization

	**Baseline**^**a**^**Mean (SD)**	**(n=81) Median (IQR)**	**6 Months Mean (SD)**	**(n=77) Median (IQR)**	**12 Months Mean (SD)**	**(n=79) Median (IQR)**	*P value	*P value (T1-T2)	*P value (T2-T3)	*P value (T1-T3)
**Variable**										
**Physician/specialist visits**	$502 (574)	$282 (398)	$356 (346)	$249 (360)	$317 (357)	$196 (307)	0.09			
**Other health care provider visits**	$4147 (5607)	$2424 (4018)	$4353 (6335)	$2115 (4058)	$4965 (6558)	$2936 (6028)	0.03	0.25	0.03	0.99
**Total health care provider visits**	$4649 (5584)	$2868 (3803)	$4709 (6341)	$2709 (4358)	$5281 (6585)	$3113 (5788)	0.01	0.03	0.05	0.99
**Use of community support services**	$781 (2008)	$0 (280)	$895 (1640)	$0 (1200)	$863 (1750)	$0 (958)	0.73			
**Diagnostic tests**	$131 (226)	$42 (186)	$153 (243)	$53 (158)	$109 (193)	$34 (123)	0.01	0.18	0.99	0.008
**Specialty Items**^**b**^	$276 (1063)	$0 (0)	$361 (1663)	$0 (8)	$140 (392)	$0 (0)	0.70			
**Medication costs**	$482 (422)	$356 (359)	$455 (383)	$356 (415)	$385 (338)	$345 (449)	<.0001	0.003	<.0001	<.0001
**Transportation within Ontario**	$474 (892)	$174 (468)	$434 (791)	$188 (379)	$373 (660)	$153 (346)	0.23			
**Work loss due to illness**	$502 (1648)	$0 (148)	$285 (698)	$0 (240)	$292 (1258)	$0 (0)	0.55			
**Work loss due to treatment**	$238 (654)	$0 (0)	$85 (348)	$0 (0)	$159 (1191)	$0 (0)	0.002	0.26	0.63	0.0006
**Government cheque received**	$1820 (3220)	$680 (2580)	$2161 (1923)	$1575 (3084)	$5620 (27817)	$1720 (3198)	0.005	0.98	0.92	<.0001
**Out of pocket expenses**	$2267 (3583)	$813 (2793)	$3875 (3151)	$3111 (4489)	$1827 (2610)	$538 (2748)	<.0001	0.001	0.0001	0.96

* Analyses using Friedman test; post-hoc testing on results where p<.05 with Sidak adjustments used to adjust the P value for multiple comparisons.

^a^ Parental report on 6 months prior to intervention.

^b^ Specialty items include special treatment (e.g. chemotherapy, antibiotics, injections, vaccinations, etc.) and supplies, aids or devices (wheelchairs, syringes, walker, etc.).

^c^ Government cheque includes all forms of government assistance, including: disability and treatment, child tax benefit, day care, workers compensation, old age security, disability pension, Ontario Guaranteed Annual Income Supplement (GAINS), Veteran’s pension, employment insurance, and social assistance [23].

^d^ Out of pocket expenses is based on the sum of community support services costs, specialty item costs, transportation costs and work loss costs.

### Health-related quality of life and family centeredness of care

Parental quality of life did not significantly change over the course of the study in any of the eight SF-36® domains (Table 5). Child quality of life improved between baseline and 6 months in two of five PedsQL**™** domains [Social domain (p=.01) and Emotional domain (p=.003)], and between baseline and 1 year in two of six CPCHILD^©^ domains [Health Standardization Section (p=.04) and Comfort and Emotions (p=.03)], but overall, the total CPCHILD^©^ score decreased between baseline and 1 year (p=.003). MPOC^©^ scores improved over the course of the study. Significantly higher parental ratings were reported at 12 months compared with baseline for three of the five domains [enabling and partnership (p=0.01), coordinated and comprehensive care (p=0.004), and respectful and supportive care (p=0.01)].

**Table 5:** Health-related quality of life for parents (SF-36®) and their children [Child Health-Related Quality of Life (PedsQL™) and Caregiver Priorities and Child Health Index of Life with Disabilities (CPCHILD®)] and Family-Centreedness of Care (MPOC©)

	**Baseline**^**a**^		**6 Months**		**12 Months**		*P value	*P value (T1-T2)	*P value (T2-T3)	*P value (T1-T3)
	**Mean (SD)**	**Median (IQR)**	**Mean (SD)**	**Median (IQR)**	**Mean (SD)**	**Median (IQR)**				
**SF-36®**^**a**^										
**Physical Functioning**	88.5 (14.8 )	95 (20)	86.7 (23.8)	100 (20)	85.1 (22.8)	95 (20)	0.79			
**Role – Physical**	75.9 (37.2 )	100 (50)	81.5 (36.1)	10 (0)	77.9 (37.4)	100 (50)	0.58			
**Bodily Pain**	71.9 (26.5)	74 (49)	74.6 (30.0)	84 (39)	76.3 (24.3)	80 (39)	0.49			
**General Health**	71.3 (21.9)	75 (33)	72.0 (22.1)	77 (25)	67.2 (25.8)	75 (25)	0.52			
**Vitality**	52.9 (25.3)	55 (40)	54.8 (22.9)	60 (30)	51.7 (22.2)	50 (30)	0.88			
**Social Functioning**	74.2 (29.9)	87.5 (37.5)	75.3 (29.6)	87.5 (50.0)	77.4 (24.7)	87.5 (37.5)	0.64			
**Role - Emotional**	77.6 (38.0 )	100.0 (33.3)	80.9 (36.8)	100.0 (0)	77.0 (41.9)	100 (33.3)	0.91			
**Mental Health**	72.7 (20.8)	80 (28)	72.7 (19.9)	76 (28)	71.4 (20.7)	76 (28)	0.98			
***PedsQL™***^***b***^										
**Average**	43.6 (14.3)	43.5 (18.1)	46.4 (18.7)	45.7 (30.2)	47.9 (20.3)	44.6 (28.8)	0.08			
**Physical**	32.4 (26.9)	21.9 (45.3)	32.9 (29.6)	25.0 (40.6)	35.2 (32.9)	22.9 (46.9)	0.16			
**Emotional**	63.4 (22.1)	70.0 (33.8)	66.1 (21.2)	65.0 (31.3)	68.7 (21.8)	70.0 (30.0)	0.02	0.003	0.83	0.48
**Social**	46.0 (19.0)	45.0 (27.5)	53.5(19.3)	50.0 (25.0)	52.7 (24.8)	50.0 (20.0)	0.006	0.011	0.98	0.09
**School**	38.5 (22.6)	35.0 (25.0)	41.8 (22.7)	40.0 (27.5)	42.8 (23.8)	40.0 (30.0)	0.97			
***CPCHILD®***^**c**^										
**Personalized Care/Activities of Daily Living**	43.4 (21.1)	44.4 (26.5)	45.7 (18.3)	44.4 (22.2)	42.4 (21.7)	37.0 (28.4)	0.48			
**Positioning, Transferring and Mobility**	50.4 (26.6)	42.4 (41.3)	49.4 (26.4)	48.6 (37.5)	48.3 (28.0)	41.7 (38.5)	0.48			
**Comfort and Emotions**	81.7 (15.8)	86.5 (21.0)	84.8 (15.4)	87.3 (17.5)	80.1 (20.6)	84.9 (21.8)	0.0006	0.21	0.002	0.03
**Communication and Social Interaction**	54.1 (21.7)	54.8 (27.4)	50.0 (25.4)	50.0 (39.9)	54.5 (25.0)	52.4 (33.3)	0.12			
**Health Standardized Section**	61.0 (19.7)	60.0 (26.7)	63.7 (17.9)	60.0 (26.7)	63.1 (20.9)	66.7 (28.3)	0.02	0.1	0.99	0.04
**Child's Overall Quality of Life**	63.1 (21.5)	60.0 (20.0)	63.9 (22.2)	60.0 (40.0)	59.7 (25.9)	60.0 (40.0)	0.10			
**Total Score**	58.2 (15.4)	57.0 (19.9)	59.0 (16.1)	57.6 (21.9)	58.4 (18.2)	56.1 (25.5)	0.0012	0.27	0.07	0.003
**MPOC©**^**d**^										
**Enabling and Partnership**	5.4 (1.4)	5.5 (1.4)	5.5 (1.6)	6.0 (1.3)	5.9 (1.4)	6.0 (1.7)	0.03	0.85	0.65	0.01
**Providing General Information**	4.2 (1.5)	4.3 (2.3)	4.8 (1.8)	5.2 (2.4)	4.8 (1.8)	5.4 (2.0)	0.03	0.1	0.09	0.08
**Providing Specific Information about the Child**	5.3 (1.5)	5.7 (2.0)	5.4 (1.7)	6.0 (2.0)	5.6 (1.6)	6.0 (1.3)	0.08			
**Coordinated and Comprehensive care**	5.5 (1.2)	5.9 (1.5)	5.9 (1.1)	6.3 (1.3)	6.0 (1.2)	6.3 (1.0)	0.002	0.13	0.42	0.004
**Respectful and Supportive Care**	5.6 (1.0)	5.8 (1.4)	6.0 (0.9)	6.0 (1.4)	6.0 (1.3)	6.2 (1.0)	0.001	0.02	0.07	0.01

* Analysis using Friedman test; post-hoc testing on results where p<.05 with Sidak adjustments used to adjust the p value for multiple comparisons.

^a^ Data available for n=79 at baseline, n=76 at 6 months, n=77 at 12 months.

^b^ For children ≥ 2 years (data available for n=54 at baseline, n=61 at 6 months, n=58 at 12 months).

^c^ For children ≥ 1 year (data available for n=65 at baseline, n=72 at 6 months, n=72 at 12 months).

^d^ Data available for n=73 at baseline, n=76 at 6 months, n=78 at 12 months.

### Qualitative perceptions of outcome and process

#### Parent reports

Parents consistently described the clinic to be highly beneficial for both their child and themselves. They highlighted the positive difference the clinic made to overall child/family care and the coordination of their child’s care across settings and specialties. Parents cited numerous examples of how with “just one call” or “email” their questions about their child’s health were answered, and/or an appointment with a specialist was organized that could help resolve their concerns. Other attributes of the clinic that parents identified as positive included: a significant reduction in travel as the clinic was closer to their home than was the tertiary center, and enhanced efficiency at the tertiary center due to their appointments with specialists being grouped, thereby requiring fewer visits. This reportedly resulted in fewer visits and thus parents benefited from savings in both time and financial costs (e.g., parking, loss of work).

#### Health care provider (HCP) reports

The majority of comments from the HCPs about the clinic centered around the helpful role of the NP in improving care for the children and families and enhancing their own quality of work life. They commented that the NP was instrumental in organizing care planning meetings that included community physicians, tertiary care specialists, allied health professionals and family members. In addition, this NP role was reported to enable the community providers to have a direct link with specialists to better understand the medical management of the child’s care. They repeatedly referred to the NP as being “key” or “the hub” for the clinic, and they suggested that the children with the most complex needs strongly benefited from the clinic.

## Discussion

Our findings support the development of complex care models focused on tertiary care-community based partnerships. While some short-term costs borne by families increased initially, likely due to the recognition of unmet needs by NPs (for example, specialty items such as a wheelchair and need for community services), families experienced long-term cost savings. Care was more likely to be delivered in community settings. Families and health care providers were highly satisfied and self-reports of family-centeredness of care improved in the absence of any substantial changes in either child or caregiver HR-QOL, despite less active subspecialty tertiary care involvement.

The results are similar to those described in the Paediatric Alliance for Coordinated Care (PACC), a study of urban primary care practices closely affiliated with tertiary care in Boston utilizing a somewhat similar model (e.g. tertiary care affiliated pediatric NP acting as case manager, development of individualized health plans); they also reported decreased hospitalization rates, decreased parental work loss, and high parental satisfaction with the intervention [31]. Our study extends the positive effects to a much broader geographic catchment by specifically demonstrating benefits of integration with suburban and rural centers with tertiary care support, and provides further evidence of cost savings in care coordination for complex pediatric populations.

There are a number of important limitations to this study. First and most importantly, the design was quasi-experimental without a control group, similar to many care coordination-type evaluations in child health [1,32]. Improved outcomes such as cost effectiveness may have therefore been related to factors other than the intervention such as the natural history of the child’s condition. However, the children in this study all had complex life-long chronic conditions, and a pattern of healthcare utilization that was increasing just prior to the intervention. Decreased utilization was noted in patterns that would be expected based on care coordination (e.g. decrease in inpatient utilization and increase in ambulatory utilization, ostensibly due to improved access to and coordination of outpatient services). Nevertheless, a controlled study design, such as a traditional randomized controlled trial or a stepped wedge trial design will be necessary to confirm our findings. Second, the study was conducted in only two centers with patient populations that had diverse diagnoses and care needs. However, the overall high prevalence of multiple diagnoses and specialists per patient, polypharmacy, complex chronic conditions, technology assistance and neurologic impairment was consistent with other complex care program descriptions [7-11]. Third, all patients recruited to participate in the study were considered to have “unmet needs”, which may have skewed the results in the direction of improvement over time. Fourth, although we assessed a relatively broad range of outcomes of relevance to high quality care, some salient measures such as disease-specific outcomes, were not assessed. Fifth, cost estimates may have been inaccurate. While we were able to collect comprehensive hospital-based costs, supplemented by parental recall of community-based costs using a standardized instrument, such data is inevitably limited by recall bias. Additionally, it is important to note that hospital costs used for this study do not reflect indirect costs, such as building maintenance and that cost effectiveness is context specific, and implications may differ in varied health systems. Some cost savings in the health care sector may have been offset by increased expenditures in other sectors (e.g. social services) and, even within the health system, savings may lead to revenue loss in a non-integrated system. Sixth, given that this study assessed implementation at one year, it does not assess long-term sustainability, including the impact on staff (e.g. NP burnout). Seventh, this evaluation of a bundled intervention did not lend itself to detailed understanding of the day-to-day functions of the NP role. It is impossible to know which parts of the intervention impacted most on outcomes, such as cost effectiveness. Specific enabling factors and barriers to implementation of this intervention cannot be identified. Finally, the lack of improvement in HR-QOL may reflect a true lack of change or rather an intrinsic limitation in the validity and sensitivity to change detectable by such instruments when applied to a diverse population of CMC.

Despite these limitations, our findings suggest that efforts to improve integration for CMC across the continuum of care have the potential for improved care with notable cost savings, albeit not equally, as savings noted in our model were largely in the tertiary care hospital. Current trends towards formalizing integration through Accountable Care Organizations (ACOs) in the United States or other global payment structures that are being utilized in different health systems that focus cost and reimbursement considerations on the patient across organizations may help to balance this issue. Moreover, ACOs may prove vital to financially integrate organizations across the care continuum, mitigating asymmetric losses in reimbursement (or revenue), and spreading financial risk. Accounting for the cost of the intervention which was $210 900 (specifically salary costs: $123 346 for a 1.0 FTE Nurse Practitioner, $46 024 for a 0.5 FTE Complex Care Social Worker, $18 408 for a 0.2 FTE Complex Care Dietician, $10 822 for a 0.1 FTE Complex Care Pharmacist, and $9 200 for a 0.1 FTE Complex Care Occupational Therapist and administrative costs of $3 100), from the perspective of costs borne by the health system, the cost savings amounted to $737 199 over a year for this population of 81 CMC, or $9101 per patient enrolled (a return on investment of 350%) without any notable increase in parental reports of health-related costs.

## Conclusions

One of the barriers to integration in pediatrics is the very nature of the epidemiology of many complex conditions which are individually uncommon and consequently geographically spread while pediatric specialty care is regionalized, necessitating both a community and a center base for many children. Systematic barriers including time necessary to form partnerships and work collaboratively and inadequate payment in current funding models continue to pose significant barriers to integration for this population. Formal partnerships between children’s hospitals and community hospitals in care coordination, together with family engagement and the primary care providers, is a promising model for complex care delivery.

## Abbreviations

(CSHCN): Children with special health care needs; (CMC): Children with medical complexity.

## Competing interests

The authors declare they have no competing interests.

## Authors’ contributions

EC conceived of the study, acquired funding, participated in its design and coordination, and drafted the manuscript; ALD performed the quantitative analysis and helped to draft the manuscript; KS participated in the design and coordination, supervised the qualitative analysis and helped to draft the manuscript; JM participated in the design and coordination, performed the qualitative analysis, and helped to draft the manuscript; DN participated in the design and coordination and reviewed the manuscript; UN participated in the design and oversaw the analysis of quality of life data; MG was the site primary investigator in Orillia; IM was the site primary investigator in Brampton; JF participated in the study design and coordination and helped to draft the manuscript. All authors read and approved the final manuscript.

## Pre-publication history

The pre-publication history for this paper can be accessed here:

http://www.biomedcentral.com/1472-6963/12/366/prepub
